# Modified strip-tillage boosts maize grain yield and water use efficiency by enhancing fine root length in Northeast China’s black soil

**DOI:** 10.3389/fpls.2026.1809158

**Published:** 2026-04-23

**Authors:** Zhiguo Yin, Qingjun Cao, Yan Ma, Eusun Han, Hao Yang, Fanli Kong, Zhengguo Cui

**Affiliations:** 1 Jilin Academy of Agricultural Sciences (Northeast Agricultural Research Center of China)/Key Laboratory of Northeast Crop Physiology Ecology and Cultivation, Ministry of agriculture and rural affairs of the People’s Republic of China, Changchun, China; 2 Faculty of Agronomy, Jilin Agricultural University, Changchun, China; 3 Department of Agroecology, Aarhus University, Tjele, Aarhus, Denmark

**Keywords:** conservation tillage, root morphology, soil moisture, spring maize, water use

## Abstract

**Introduction:**

Soil moisture deficit limits crop productivity in rain-fed areas, and root system architecture and plasticity are closely related to water uptake and utilization.

**Methods:**

This study explored maize grain yield, soil moisture, root architectural plasticity, and their relationships with water use efficiency under modified strip-tillage (MST), no-tillage (NT), and conventional tillage (CT, as control) practices using two-year (2023–2024) field experiments in the black soil region of the Songliao Plain, China.

**Results:**

The results showed that compared with conventional tillage, grain yield increased by 13.32%–17.15% under no-tillage and 11.89%–16.06% under modified strip-tillage. Tillage practices significantly affected silking-stage root density (root length density, etc.), root morphology, and root diameter classes (e.g., < 0.5 mm). These root-related indices were higher under MST and NT than under CT, with no significant difference between MST and NT. Specifically, MST increased fine root length (root diameter, RD < 0.5 mm) by 93.23%–95.22% (vs. CT) and 39.90%–66.15% (vs. NT). The two conservation tillage treatments (MST and NT) increased the average soil moisture in the 0–40 cm layer during the growing season by 12.80%–31.43% and 11.44%–31.48%, respectively, compared with CT. Additionally, compared with CT, MST reduced water consumption by 1.95%–2.41% and increased water use efficiency by 16.33%–18.27%. Structural equation modeling showed that conservation tillage improved grain yield by optimizing root traits (e.g., root density, root morphology) and water use efficiency. MST performed better in these aspects, with its yield benefit attributed to the coordinated regulation of these traits.

## 1 Introduction

Maize (*Zea mays L.*) is a crucial food, feed, and energy crop worldwide, and the black soil region in Northeast China, a primary production base for spring maize in China, contributing approximately 30% of the national total maize output (Yan and Chen, 2025). It is a typical rain-fed agricultural region characterized by insufficient rainfall that fails to meet the water requirements for normal maize growth and production, making improving the utilization efficiency of natural rainfall a top priority for agricultural production in this area (Zhang et al., 2022; Gao et al., 2024); besides such water scarcity, the region has long relied on intensive farming practices at the expense of soil health to address drought stress and sustain maize yields, and coupled with excessive land reclamation under the conventional ridge-furrow cropping system, these practices have led to an overall 50%–70% reduction in soil organic matter (SOM) content from 1950 to 2020, significantly accelerating farmland ecosystem degradation (Wu et al., 2024), with black soil resources projected to be completely depleted within approximately 113 years if this trend is not curbed (Wang H. et al., 2022), thus making the conservation of black soil resources a strategic priority both in China and globally (Rui et al., 2025).

Conservation tillage is an agricultural tillage system characterized by the core technical features of straw mulching and minimal soil disturbance (Hobbs et al., 2008). Specifically, this involves retaining crop residues on the soil surface to protect the soil while reducing the intensity and frequency of mechanical soil disturbance, thereby maintaining soil structure and biological activity (Lal, 1997; Six et al., 2000). Extensive studies have shown that conservation tillage plays a pivotal role in alleviating soil water shortage and wind erosion, thereby supporting its adoption as a sustainable modern agricultural practice (Van Wie et al., 2013; Chen et al., 2015). Within conservation tillage systems, straw mulching has been widely validated as an effective strategy for improving water use efficiency (WUE). The key mechanisms include the reduction of soil water loss through suppressed evaporation and the enhancement of soil water storage and infiltration resulting from long-term no-tillage or reduced tillage practices combined with residue retention (Du et al., 2022; Zhang et al., 2022; Li et al., 2025). For example, a 20-year field experiment in the black soil region of Northeast China showed that, compared with conventional tillage, conservation tillage increased the average soil moisture in the 0–5 cm layer by approximately 15%; primarily due to reduced soil evaporation (Liu et al., 2025). Similarly, in the Southern Nations, Nationalities, and Peoples’ Region (SNNPR) of Ethiopia, conservation tillage increased soil water content in the 0–20 cm topsoil horizon by a range of 8%–57% relative to conventional tillage, which is driven by reduced soil disturbance and improved soil structure (i.e., decreased bulk density and increased porosity) (Bekele et al., 2022). In addition, because tillage-induced changes in soil thermal, physical, and moisture conditions are first perceived by the belowground system (Cabal et al., 2021; Du et al., 2022), crop roots represent a key interface through which conservation tillage and modified strip-tillage influence crop water uptake, growth, and yield formation. As a core functional organ of crops, the root system primarily functions to absorb and transport water and nutrients (Yin et al., 2024). In black soil regions, conservation tillage can improve soil aggregate structure, optimizing the crop root growth environment, and enhancing crop access to deep soil moisture (Sha et al., 2024); such improvements to the farmland environment further enhance root system establishment, including root density distribution (Ma et al., 2023) and morphological improvement (Heinen et al., 2025). Compared to conventional tillage practices, maize grown under conservation tillage exhibits deeper root systems, greater root density, leading to enhanced resource acquisition capacity (Roldán et al., 2003) and crop yield (Himmelbauer et al., 2012).

While conservation tillage offers notable advantages in water conservation and soil erosion mitigation as elaborated earlier, large-scale no-tillage with full straw mulching faces practical challenges in alpine and cold regions (Wang et al., 2024). Specifically, the residual undecomposed straw from the previous year forms a surface mulch during sowing, which lowers soil temperatures in early spring. This not only delays sowing but also impairs seedling establishment (e.g., uneven germination and insufficient effective plant density), ultimately leading to significant yield losses (Zhang et al., 2015; Wang et al., 2024). Additionally, long-term no-tillage practices can reduce root dry weight, length, and surface area due to increased soil bulk density and higher penetration resistance—factors that hinder root penetration into the subsoil and limit the exploration of soil resources, for example, in maize (Fiorini et al., 2018). Therefore, conservation tillage integrating straw mulching with modified strip-tillage has emerged as an effective strategy to alleviate low-temperature stress and yield losses associated with no-tillage. Modified strip-tillage involves localized soil disturbance confined to narrow planting strips, while crop residues are retained on the soil surface between rows; this approach improves seedbed conditions and early-season soil thermal and physical properties without compromising the soil protection provided by residue mulching (Chen et al., 2021; Zhang et al., 2024; Liu et al., 2025). In recent years, this improved practice has been increasingly popularized in the black soil regions of Northeast China (Chen et al., 2021). Despite the clear effects of these tillage practices on crop growth, no consistent conclusion has been reached regarding the growth and development of crop roots under conservation tillage systems. In fact, root development itself is regulated by multiple factors, including soil type, soil physical and chemical properties, climatic conditions, geographical locations, and tillage management practices (Watt et al., 2006; Han et al., 2015, 2024). On the other hand, ridge tillage, a conventional tillage practice historically adopted in the black soil regions of Northeast China (Xu et al., 2018), involves intensive soil disturbance through repeated rotary tillage and ridging after straw removal. This practice not only exacerbates soil water loss and impairs seedling establishment (Xu et al., 2018; Zhang et al., 2024) but also leads to soil compaction and reduced soil permeability with long-term use, ultimately limiting soil water storage and precipitation use efficiency (Kuncoro et al., 2014; Lin et al., 2022).

Against this background, this study aimed to compare the effects of conventional tillage, no-tillage, and modified strip-tillage on soil moisture dynamics, root morphological traits, water use efficiency, and grain yield of maize in the rain-fed black soil region. The specific objectives were to clarify the regulatory mechanisms of modified strip-tillage on soil water storage and root system development, and to quantify their combined effects on maize yield formation.

## 2 Materials and methods

### 2.1 Characteristics of the study area

**Figure 1:**
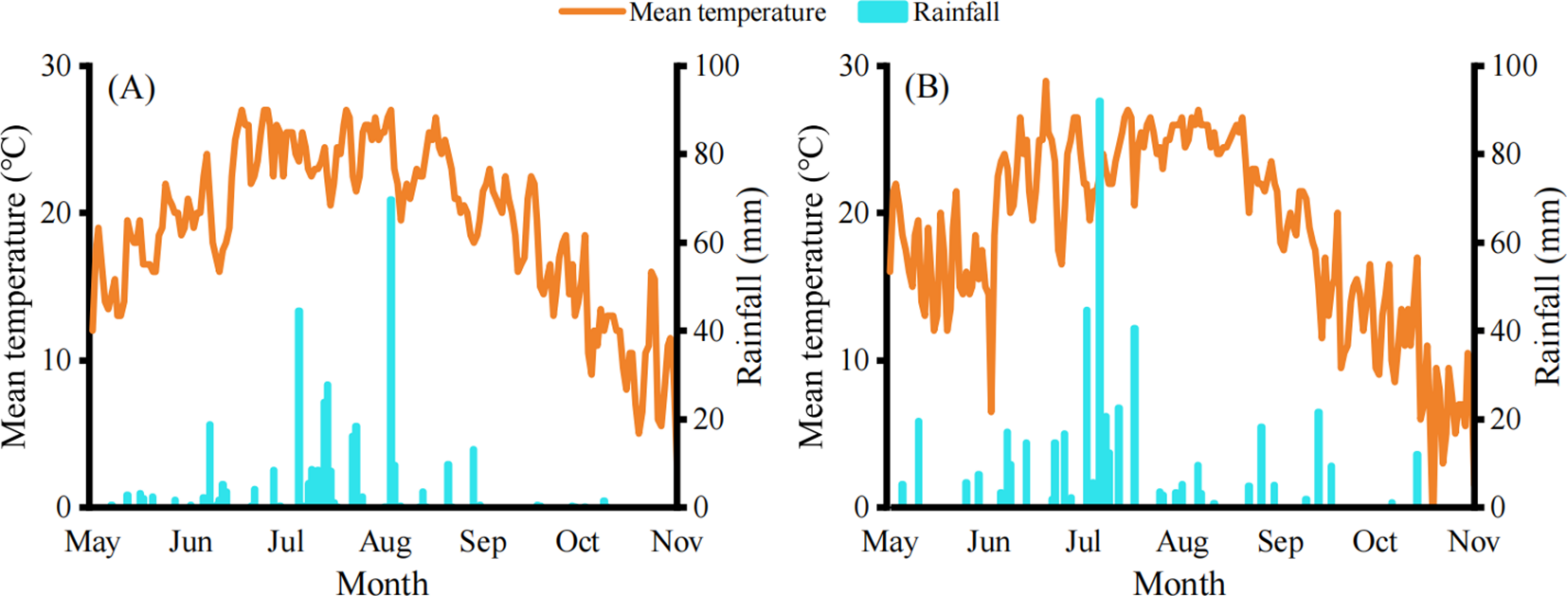
Daily mean temperature (°C, orange line) and rainfall (mm, blue bars) at the study site in 2023 **(A)** and 2024 **(B)**.

Field experiments were carried out at the Caijia Experimental Station, affiliated with the Soil Health Research Group of Jilin Academy of Agricultural Sciences (JAAS) (123°57’46”E, 43°28’36” N) in 2023–2024. The experimental site, located in Northeast China, features typical black soil (IUSS Working Group WRB, 2014). Prior to the experiment initiation, the basic physical and chemical properties of the 0–20 cm soil layer were as follows: Soil bulk density was 1.51 g/cm³, soil organic matter content 29.73 g/kg, hydrolyzable nitrogen 166.4 mg/kg, available phosphorus 32.14 mg/kg, available potassium 208 mg/kg, total nitrogen 1.45 g/kg, total phosphorus 0.57 g/kg, total potassium 22.76 g/kg, and pH value 6.46. The rainfall and daily average temperature during the maize growing seasons at the experimental site are presented in **Figure 1**.

### 2.2 Experimental design and management

Three cultivation treatments were established in this experiment: (1) Modified Strip-tillage with partial straw mulching (MST): After maize harvest, straw was crushed and surface-mulched. A straw row cleaner was used to gather straw from the predetermined seedbed zones onto the fallow strips, followed by strip tillage to form standardized seedbeds (width of 50 ± 5 cm and depth of 5–7 cm). In the subsequent spring, wide-narrow row sowing was performed in the seedbeds using a Specialized Wide-Narrow Row No-Till Maize Seeder, with a narrow row spacing of 40 cm and a wide row spacing of 80 cm. Throughout the growing season, straw was fully mulched between the wide rows. (2) No-tillage with straw mulching and incorporation (NT): After harvest, maize straw was crushed and surface-mulched without any tillage operations. In the following spring, wide-narrow row sowing was conducted using a no-tillage planter as in modified strip-tillage, with subsequent procedures identical to those of the modified strip-tillage treatment. (3) Conventional tillage (CT): After maize harvest, straw was completely removed from the field. In the next spring, rotary tillage (depth: 15 ± 2 cm) was carried out, followed by ridging to form wide ridges (ridge top width: 50 cm; ridge top height: 15 ± 2 cm). Sowing was then performed using a no-tillage planter, with other management practices consistent with the modified strip-tillage treatment. (See **Supplementary Figure**).

A randomized complete block design (RCBD) was adopted, with three replicates per treatment. Each experimental plot covered an area of 600 m² (50 m long × 12 m wide), the same maize cultivar (hybrid F15A), provided by Xuanyu Seed Industry Co., Ltd. (Jilin Province), was used for both experimental years (2023–2024). Planting density and fertilization rate were consistent across all treatments. The fertilization scheme was as follows: Nitrogen (N) was applied at 240 kg·hm^-2^, phosphorus pentoxide (P_2_O_5_) at 90 kg·hm^-2^, and potassium oxide (K_2_O) at 100 kg·hm^-2^. All fertilizers were applied as a one-time basal addressing synchronously with sowing. The planting density was (5.5 ± 0.1) × 10^4^ plants·hm^-2^. Uniform sowing was conducted on May 12^th^, and harvesting on October 8^th^ for both years. Three days after sowing, a mixed formulation of acetochlor and atrazine was sprayed for pre-emergence weed control. Chlorantraniliprole (produced by Syngenta AG) was applied at the 7-leaf stage and silking stage of maize to control *Ostrinia furnacalis* (Guenée), respectively.

### 2.3 Root measurement

**Figure 2:**
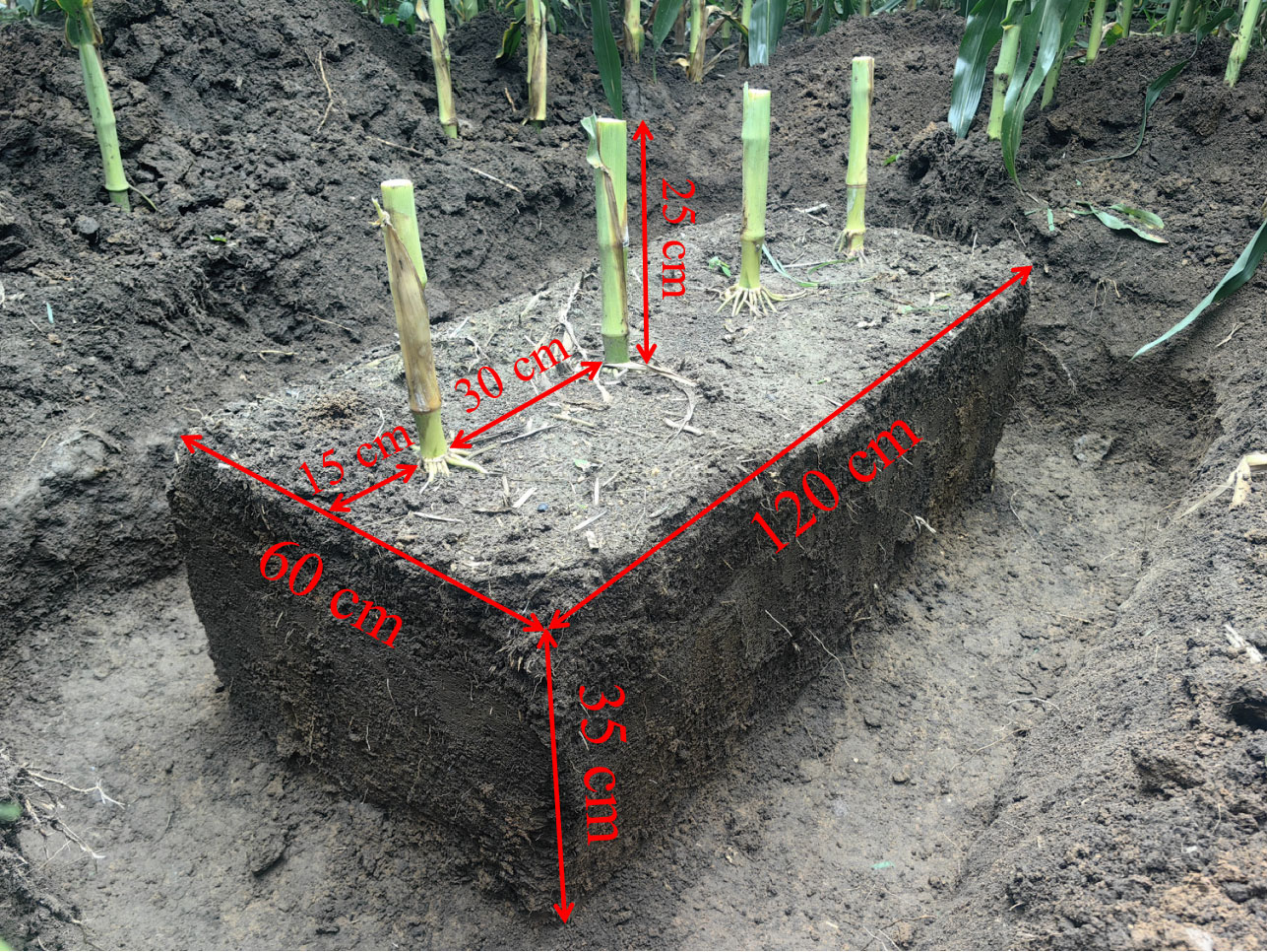
A photo illustrating the excavation for root samples at the study site (Caijia Experimental Station, JAAS) after harvest on 2024-08-20.

Root excavation was performed following the method described by Shao et al. (2019). At the maize flowering stage, soil monoliths (120 cm × 60 cm × 35 cm; length × width × depth) were exposed by cutting down surrounding area (see **Figure 2**). For each sample unit, 25 cm of the aboveground portion was retained from the base of the plant, with four consecutive plants sampled separately. The average soil volume (SV) per single sample was 6.3 × 10^4^ cm^3^ (**Figure 2**). Subsequently, the excavated roots were soaked in clean water for 12 h at room temperature (20-25°C) and then thoroughly rinsed to remove adhering soil particles. The clean root samples were photo-scanned using MRS-9600TFU2L scanner (Bangyi Precision Instrument Co., Ltd., Shanghai, China) with 300 dots per inch (DPI) resolution. WinRhizo software (Regent Instruments INC., Quebec, Canada) was used to determine root parameters, including root length (RL; cm), root surface area (RSA; cm^2^), root volume (RV; cm^3^), root mean diameter (RMD; mm), root tip number (RT), root fork number (RF), and root crossing number (RC). Root length categorized by root diameter classes (root diameter, RD < 0.5, 0.5–2.0, 2.0–3.0, 3.0–5.0, and ≥ 5.0 mm). After scanning, the roots were placed in kraft paper bags and oven-dried at 80°C for 36 hours. Root dry weight (RDW; g) was then measured and recorded. Additionally, the characteristics of maize roots per unit volume were analyzed, including root length density (RLD; cm cm^-3^), root biomass (RB; mg cm^-3^), specific root length (SRL; cm g^-1^). The calculation formula for these parameters are as follows: All variables are as previously defined.

(1) $$RLD\;(cm\;cm^{-1})=\frac{RL}{SV}$$

(2) $$RB\;(mg\;cm^{-3})=\frac{RDW}{SV}$$

(3) $$SRL\;(cm\;g^{-1})=\frac{RL}{RDW}$$

### 2.4 Soil moisture content and soil water storage determination

Soil moisture content (SMC; %) was determined using the oven-drying method. Samples were collected at soil depths of 0–10, 10–20, 20–30, 30–40, 40–60, 60–80, and 80–100 cm at three key growth stages of maize: pre-sowing, flowering, and post-harvest stage. Subsequently, the total soil water storage (SWS; mm) in the 0–100 cm soil layer was further calculated. Samples were taken from ridge tops and furrows in CT treatment. For no-tillage (NT) and modified strip-tillage (MST) treatments, samples were collected from seedbeds without straw mulch and from fallow areas with straw mulch, respectively. The calculation formula is:

(4) $$SMC\;(\%)=\frac{w_{s}-w_{g}}{W_{g}}\times 100\%$$

In the equation, *Ws* represents the weight of wet soil (g), while *Wg* indicates the weight of dry soil (g).

(5) SWS (mm)=d×r×w×10

In the formula, *d* is soil thickness (cm), *r* is soil bulk density (g/cm^3^), *w* is soil water content (%), and 10 is the conversion coefficient.

### 2.5 Maize grain yield

At mature stage, 2 rows (not sampled) were harvested in each plot, and dried naturally after harvest. After threshing, the yield was measured (according to the standard water content of 14%). After harvest, 10 ears were randomly selected from each plot, and the row number, grain number per row and 1000 grain weight were measured.

### 2.6 Water use efficiency and precipitation use efficiency

The calculation formula for water consumption (WC) of maize during the growing season is as follows:

(6) WC (mm)=SWS1+P+U+Q+F−SWS2

In the formula, where *WC* denotes the water consumption of maize during the growing period (mm); *SWS_1_* and *SWS_2_* represent pre-sowing and post-harvest soil water storage (mm), respectively; P is the precipitation during the crop growing period (mm); *U* is the groundwater recharge (mm); *Q* is the surface runoff (mm); and *F* is the soil water leakage (mm). In the high-latitude semi-arid rain-fed agricultural region, rainfall is limited, deep percolation rarely occurs, and surface runoff is negligible. Moreover, the experimental site has an average altitude of approximately 200 m with a deep groundwater table. Therefore, these factors are generally neglected in the calculation (Moiwo and Tao, 2015). Based on this, Equation (7) can be simplified as follows:

(7) $$WUE\;(kg\;ha^{-1}\cdot mm^{-1})=\frac{GY}{WC}$$

In the formula, *GY* represents grain yield (kg/hm²), *WC* signifies water consumption of maize during the growing period (mm).

Precipitation use efficiency (PUE; kg·ha^−^¹·mm^−^¹) was defined as the ratio of crop grain yield to the total precipitation during the growing season.

(8) $$PUE\;(kg\;ha^{-1}\cdot mm^{-1})=\frac{GY}{P}$$

In the formula, GY represents grain yield (kg/hm²), P indicates growing season precipitation (mm). Rainfall data were continuously recorded via positioning observation by the automatic weather station (AWS) deployed in the experimental area.

### 2.7 Statistics and analysis

A two-way analysis of variance (ANOVA) was performed using SPSS 26.0 (IBM Corporation, Armonk, New York, USA) to evaluate the effects of different tillage year (Y), tillage practices (TP) and their interaction (Y×TP). Duncan’s multiple range test was determined significant differences among treatments at the 0.05 probability level (*p* < 0.05). Graphs were plotted using Origin 2021(OriginLab Corporation, Northampton, Massachusetts, USA).

Other statistical analyses were performed using R version 4.3.1 (R Core Team, 2023). Following Z-score standardization, Bartlett’s test of sphericity was highly significant (*p* < 0.001), confirming the suitability of data for principal component analysis (PCA). PCA was conducted using the FactoMineR package with varimax rotation. The factoextra package was used to extract loading coefficients, and the first principal component (PC1) was extracted to construct comprehensive indices. A random forest model was constructed using the randomForest package with 500 decision trees, mtry = 2, and a random seed of 50 to ensure reproducibility, to assess the importance of variables in influencing maize yield. Variable importance was evaluated by IncNodePurity and %IncMSE, with significance determined using 100 permutation tests. Model performance was verified by R², mean squared error (MSE), and root mean squared error (RMSE). Piecewise structural equation modeling (piecewise SEM) was performed with the piecewiseSEM package to explore the effects of variables on maize yield. This model is suitable for small-sample datasets; model fit was evaluated using Fisher’s C statistic, P-value, and Akaike information criterion (AIC), and the model included only measured observed variables with no latent variables. Finally, all visualizations were generated using the ggplot2 package.

## 3 Results

### 3.1 Grain yield and ear traits

Maize grain yield was significantly (*p* < 0.01) affected by tillage practices and year of maize growth (*p* < 0.05; see **Table 1**). Compared with conventional tillage, the grain yield under conservation tillage practices of no-tillage and modified strip-tillage increased by 11.89% and 17.15%, respectively, in 2023. In 2024, the grain yield under no-tillage and modified strip-tillage were correspondingly increased by 13.32% and 16.06%, respectively.

**Table 1:** Maize grain yield and ear traits under different tillage practices.

Year	Tillage practices	Grain yield (kg/hm²)	Ear length (cm)	Rows per ear	Kernels per row	100-grain weight (g)
2023	CT	10603.56 ± 133.88b	18.32 ± 0.32c	15.07 ± 0.70b	36.77 ± 0.61c	32.54 ± 0.46b
	NT	12421.85 ± 205.75a	20.62 ± 0.33a	15.93 ± 0.42ab	43.03 ± 1.02a	42.13 ± 1.14a
	MST	11864.44 ± 630.69a	19.23 ± 0.40b	16.33 ± 0.50a	38.97 ± 1.10b	38.52 ± 3.01a
2024	CT	11040.70 ± 293.67b	18.78 ± 0.58b	16.73 ± 0.42b	39.50 ± 1.47b	39.64 ± 0.90b
	NT	12511.82 ± 505.92a	20.78 ± 0.68a	17.27 ± 0.12ab	41.40 ± 0.26a	41.31 ± 0.30a
	MST	12813.85 ± 491.15a	20.83 ± 0.69a	17.40 ± 0.20a	41.83 ± 0.45a	41.49 ± 0.32a
Source of variance (p-value)						
Year (Y)		*	**	***	**	***
Tillage practices (TP)		***	***	**	***	***
Y×TP		ns	ns	ns	**	**

CT, conventional tillage; NT, no-tillage; MST, modified strip-tillage. Different letters indicate significant differences (Duncan’s test, *p* < 0.05). *p < 0.05; **p < 0.01; ***p < 0.001; ns, no significant difference. Mean ± S.D (n=3).

In terms of maize ear traits, conservation tillage practices significantly enhanced key traits such as ear length, number of rows per ear and number of kernels per row. In 2023, compared with conventional tillage, the ear length, rows per ear, kernels per row, and 100-grain weight under no-tillage increased by 12.55%, 5.71%, 17.02%, and 29.47%, respectively; under modified strip-tillage, these traits increased by 4.97%, 8.36%, 5.98%, and 18.38%, respectively. In 2024, ear length under no-tillage and modified strip-tillage was 10.65% and 10.92% higher than that under conservation tillage, respectively; rows per ear increased by 3.23% and 4.00%, respectively, while kernels per row increased by 4.81% and 5.90%, respectively, and 100-grain weight increased by 4.21% and 4.67%, respectively. Consistent with the trend observed for maize grain yield, all the aforementioned ear traits were significantly higher in 2024 than in 2023 across all treatments.

### 3.2 Root density and architectural traits

In this section, Equations 1, 2 were used to calculate relevant indicators. As shown in **Table 2**, tillage practice as a factor significantly influenced root density and architectural traits, and the effect was attributed only between conventional tillage (i.e., conventional tillage) and conservation tillage practices (i.e., no-tillage and modified strip-tillage) as revealed by postdoc test.

**Table 2:** Root density and architectural traits of maize at the silking stage.

Year	Tillage practices	Root length density (cm cm^-3^)	Root biomass (mg cm^-3^)	Root surface area (cm^2^)	Root volume (cm^3^)	Root tip number (k plant^-1^)	Root fork number (k plant^-1^)	Root crossing number (k plant^-1^)	Root-shoot ratio
2023	CT	0.15 ± 0.00b	0.25 ± 0.01b	5090.75 ± 505.44b	434.16 ± 43.77b	43.63 ± 5.26b	23.73 ± 2.16b	3.72 ± 0.75b	0.048 ± 0.001b
	NT	0.20 ± 0.01a	0.30 ± 0.02a	6782.38 ± 925.13a	664.75 ± 70.74a	49.95 ± 2.45b	26.71 ± 2.31b	4.01 ± 0.72b	0.053 ± 0.003a
	MST	0.22 ± 0.03a	0.33 ± 0.02a	7579.45 ± 571.50a	719.57 ± 99.40a	65.37 ± 8.84a	42.18 ± 9.07a	5.61 ± 0.38a	0.057 ± 0.003a
2024	CT	0.15 ± 0.00b	0.20 ± 0.02b	5259.15 ± 587.18b	535.28 ± 17.86b	40.74 ± 3.53b	19.66 ± 1.27b	3.37 ± 0.22b	0.028 ± 0.002b
	NT	0.19 ± 0.01a	0.24 ± 0.02a	7465.35 ± 898.62a	764.04 ± 124.62a	50.45 ± 4.26a	22.92 ± 1.42b	3.69 ± 0.64b	0.032 ± 0.002a
	MST	0.19 ± 0.02a	0.25 ± 0.03a	7898.34 ± 1089.05a	849.83 ± 143.22a	56.15 ± 5.29a	27.56 ± 2.88a	4.80 ± 0.40a	0.033 ± 0.003a
Source of variance (p-value)									
Year (Y)		ns	***	ns	**	ns	***	ns	***
Tillage practices (TP)		***	***	**	*	***	***	***	***
Y×TP		ns	ns	ns	ns	ns	ns	ns	ns

CT, conventional tillage; NT, no-tillage; MST, modified strip-tillage. Different letters indicate significant differences (Duncan’s test, *p* < 0.05). *p < 0.05; **p < 0.01; ***p < 0.001; ns, no significant difference. Mean ± S.D (n=3).

In 2023, the root density (i.e., root length density, root biomass, root surface area, and root volume) and root-shoot ratio under no-tillage increased by 33.33%, 20.00%, 33.23%, 53.11%, and 10.42%, respectively, as compared to conventional tillage. Correspondingly, the root density indices under modified strip-tillage increased by 26.67%, 25.00%, 50.18% and 58.76%. Meanwhile, architectural traits (i.e., root tip number, root fork number, and root crossing number) and root-shoot ratio increased by 37.83%, 40.18%, 42.43%, and 17.86%, in 2024, respectively.

### 3.3 Root morphology and root diameter classes

**Figure 3:**
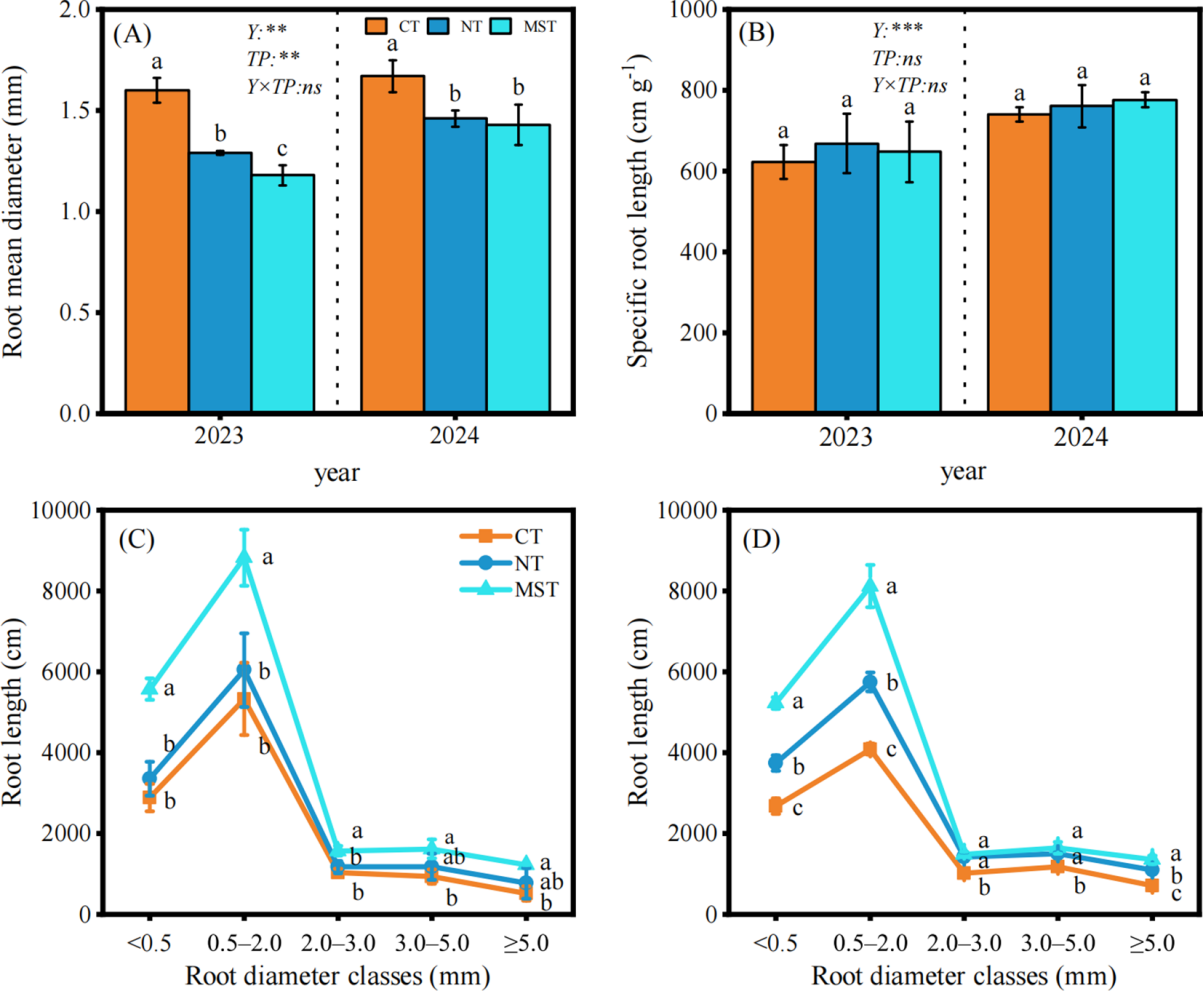
Root morphology and diameter class distribution under different tillage practices (CT, conventional tillage; NT, no-tillage; MST, modified strip-tillage) in 2023 and 2024. **(A)** Root mean diameter (mm); **(B)** Specific root length (cm g^-1^); **(C)** 2023 root length (cm) distribution; **(D)** 2024 root length (cm) distribution. Different letters indicate significant differences, **p < 0.01; ***p < 0.001; ns, no significant difference. (Duncan’s test, *p* < 0.05; mean ± SD, n=3).

**Figure 3** shows the changes in root morphology. Root mean diameter (RMD; mm, **Figure 3A**) was significantly affected by tillage practice and year – without interactions. Specific root length (SRL; cm g^-1^, calculated by Equation 3, **Figure 3B**) showed no effect of tillage practice. Postdoc test on root diameter revealed significant differences between conventional tillage practice and no-tillage and modified strip-tillage, in which root diameter decreases towards the two conservation tillage practices. Although not significant, there was a tendency for specific root length to increase from conventional tillage to conservation practices.

Root length (RL; mm, **Figures 3C, D**) was further analyzed for their diameter class distribution between the sizes of < 0.5 mm, 0.5–2.0 mm, 2.0–3.0 mm, 3.0–5.0 mm, and ≥ 5.0 mm. Consistent results across both experimental years showed that the root length of the three fine root diameter classes (root diameter, RD < 0.5 mm, 0.5–2.0 mm, and 2.0–3.0 mm) measured under the modified strip was significantly higher than those under no-tillage treatment (*p* < 0.05), and both modified strip-tillage and no-tillage had higher root length values than the conventional tillage treatment (*p* < 0.05). In contrast, across the two larger root diameter classes (RD 3.0–5.0 mm and ≥5.0 mm), root length under modified strip-tillage was significantly higher than that under no-tillage and CT (*p* < 0.05), while no significant difference was observed between no-tillage and conventional tillage (*p* > 0.05).

### 3.4 Soil moisture content distribution in various soil layers

**Figure 4:**
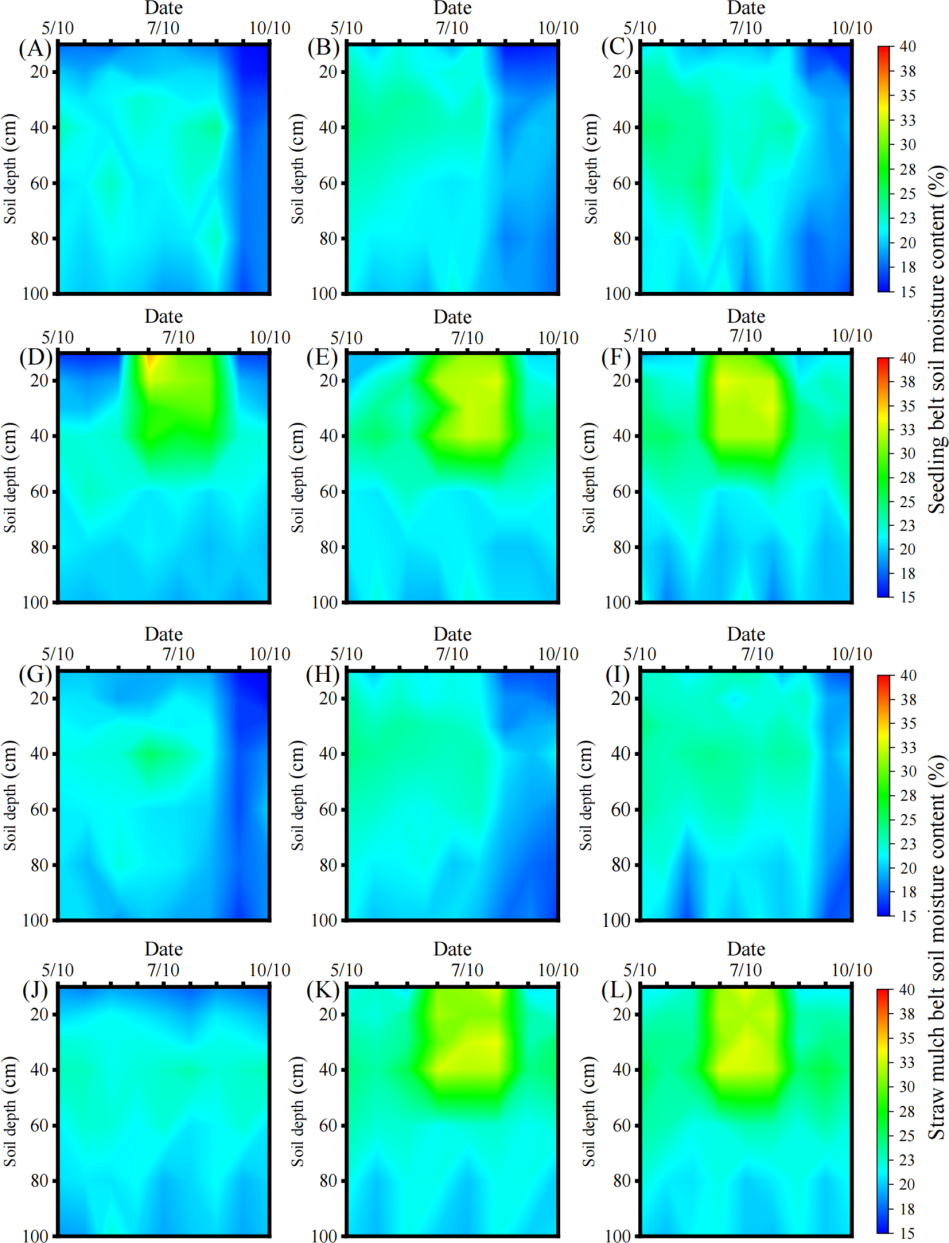
Soil moisture content in the 0–100 cm soil profile throughout the maize growing season under different tillage practices. All panels are arranged left to right as conventional tillage, no-tillage, modified strip-tillage. **(A–C)** Seedling belt soil moisture content in 2023; **(D–F)** Seedling belt soil moisture content in 2024; **(G–I)** Straw mulch belt soil moisture content in 2023; **(J–L)** Straw mulch belt soil moisture content in 2024. Mean ± SD, n=3.

**Figure 4** illustrates the variations in soil moisture content (SMC; %, calculated by Equation 4) under different tillage practices in the 0–100 cm soil depth across various maize growth stages. In 2023, within the seedling belt (**Figures 4A–C**), at pre-sowing (May 10), the soil moisture content in the 0–40 cm soil layer under no-tillage and modified strip-tillage treatments exhibited average increases of 12.98% and 11.77%, respectively, compared with conventional tillage. At the silking stage (July 10), significant differences (*p* < 0.05) were only observed in the 0–20 cm soil layer, where no-tillage and modified strip-tillage increased soil moisture content by an average of 9.31% and 6.99%, respectively, relative to conventional tillage. At post-harvest (October/10), the 0–30 cm soil layer under no-tillage and modified strip-tillage had average soil moisture content increments of 8.90% and 10.32%, respectively, compared with conventional tillage.

In 2024 (**Figures 4D–F**), relative to conventional tillage, no-tillage increased the soil moisture content in the 0–40 cm soil layer by an average of 11.10%, 14.40%, and 14.02% at three key growth stages (pre-sowing, flowering, and post-harvest), respectively. For modified strip-tillage treatment, the corresponding average increases at the same soil layer and stages were 13.46%, 15.51%, and 15.41%.

In the straw mulch belt in 2023 (**Figures 4G–I**): at pre-sowing, the soil moisture content in the 0–40 cm soil layer under modified strip-tillage and no-tillage showed average increases of 9.68% and 7.96%, respectively, compared to conventional tillage. At the silking stage, significant differences in soil moisture content were only observed in the 0–20 cm soil layer (*p* < 0.05), with modified strip-tillage and no-tillage exhibiting average increments of 9.99% and 8.03%, respectively, compared to the conventional tillage. At post-harvest, soil moisture content in the 0–40 cm soil layer under modified strip-tillage and no-tillage increased by an average of 14.76% and 13.76%, respectively, compared with conventional tillage. In 2024 (**Figures 4J–L**), across three key growth stages (pre-sowing, silking, and post-harvest), no-tillage and modified strip-tillage increased soil moisture content in the 0–40 cm layer by an average of 9.83% and 11.26% at pre-sowing, 16.87% and 19.10% at silking, and 12.14% and 12.65% at post-harvest. For the 40–100 cm soil layer, no statistically significant differences in soil moisture content were observed among the tillage practices in both the seedling belt and the straw mulch belt (*p* > 0.05).

### 3.5 Soil moisture storage, soil water storage and utilization

In this section, Equations 5–8 were used to calculate relevant indicators. Based on the two-year experimental data (**Table 3**), both no-tillage and modified strip-tillage treatments significantly increased pre-sowing soil water storage and post-harvest soil water storage compared with conventional tillage (*p* < 0.05). In 2023, pre-sowing soil water storage under no-tillage and modified strip-tillage increased by 6.86% and 7.70%, respectively, compared to conventional tillage, while post-harvest soil water storage rose by 6.01% and 9.61%, respectively. During the growing season, water consumption under the modified strip-tillage decreased by 2.41% compared to conventional tillage, whereas no significant difference in water consumption was observed between no-tillage and conventional tillage (*p* > 0.05). Additionally, precipitation utilization efficiency and water use efficiency under no-tillage and modified strip-tillage treatments were 16.03% and 18.48% higher than those under conventional tillage, respectively; the corresponding increases under modified strip-tillage were 13.28% and 16.33%.

**Table 3:** The effect of different tillage practices on maize water use.

Year	Tillage practices	Pre-sowing soil water storage (mm)	Growing season precipitation (mm)	Post-harvest soil water storage (mm)	Water consumption (mm)	Precipitation utilization efficiency (kg ha^-1^·mm^-1^)	Water use efficiency (kg ha^-1^·mm^-1^)
2023	CT	295.61 ± 2.59b	343.50a	246.00 ± 5.95b	399.13 ± 3.09a	30.87 ± 0.39b	26.51 ± 0.52b
	NT	315.89 ± 10.47a	343.50a	260.78 ± 5.00a	393.97 ± 6.86ab	35.82 ± 1.18a	31.41 ± 1.48a
	MST	318.38 ± 0.33a	343.50a	269.63 ± 5.64a	389.51 ± 3.17b	34.97 ± 0.37a	30.84 ± 0.76a
2024	CT	294.94 ± 3.08b	460.30a	290.68 ± 3.53b	468.24 ± 3.13a	23.99 ± 0.64b	23.64 ± 0.66b
	NT	312.37 ± 3.57a	460.30a	309.92 ± 0.50a	462.03 ± 3.24ab	27.18 ± 1.10a	27.03 ± 0.89a
	MST	313.72 ± 7.34a	460.30a	311.74 ± 3.26a	459.11 ± 3.04b	27.84 ± 1.07a	27.96 ± 0.90a
Source of variance (p-value)							
Year (Y)		ns	ns	***	***	***	***
Tillage practices (TP)		***	ns	***	**	***	***
Y×TP		ns	ns	ns	ns	ns	ns

CT, conventional tillage; NT, no-tillage; MST, modified strip-tillage. Different letters indicate significant differences (Duncan’s test, *p* < 0.05). ** *p* < 0.01; ***p < 0.001; ns, no significant difference. Mean ± S.D (n=3).

In 2024, pre-sowing soil water storage under no-tillage and modified strip-tillage increased by 5.91% and 6.37%, respectively, compared to conventional tillage, and post-harvest soil water storage increased by 6.62% and 7.25%, respectively. For growing season water consumption, modified strip-tillage reduced water consumption by 1.95% relative to conventional tillage. Correspondingly, precipitation utilization efficiency under no-tillage and modified strip-tillage was 13.30% and 16.05% higher than that under conventional tillage, respectively, while water use efficiency improved by 14.34% and 18.27%, respectively, with the differences reaching an extremely significant level (*p* < 0.01).

### 3.6 Relationships between grain yield, root morphological traits, and soil water conditions

**Figure 5:**
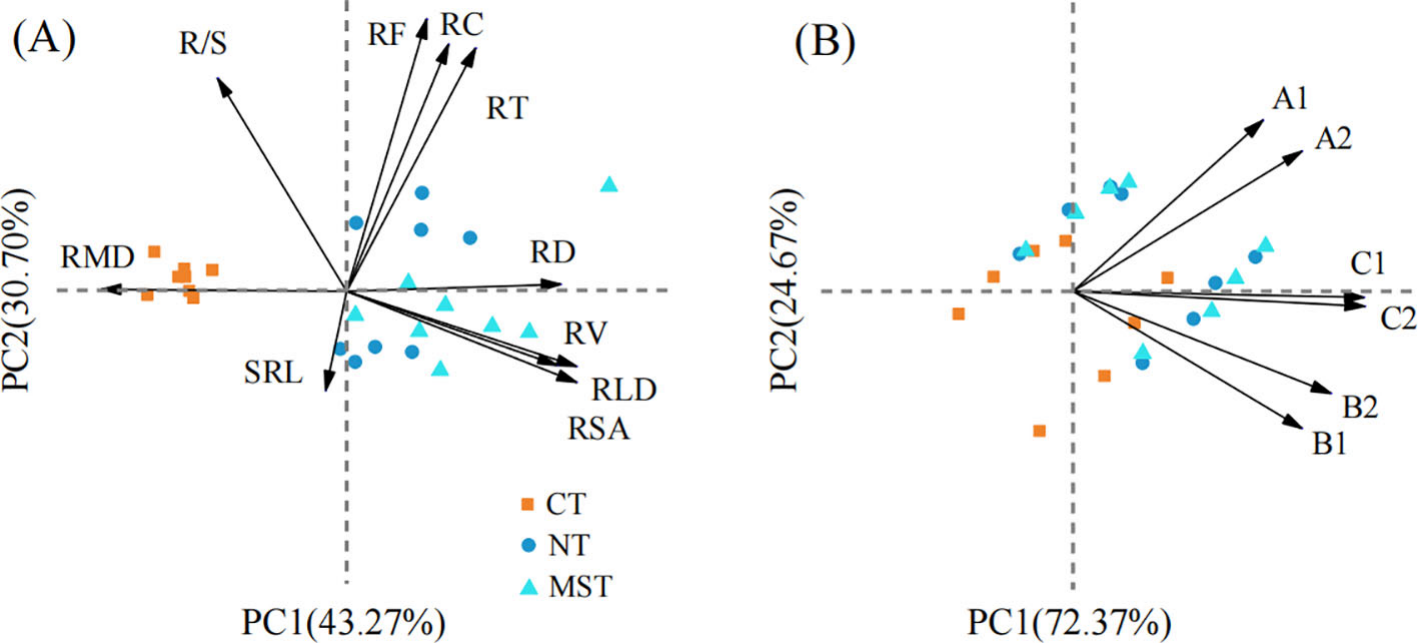
Results of principal component analysis of root morphology index (RMI, **A**) and soil moisture content index (SMCI, **B**). In panel **(B)**, A1 and A2 represent soil moisture content in the seedling belt and straw mulch belt at the pre-sowing stage, respectively; B1 and B2 represent those in the corresponding belts at the silking stage; C1 and C2 represent those in the corresponding belts at the post-harvest stage.

To comprehensively characterize the correlation between complex root morphological traits and soil water conditions under different tillage practices, we performed principal component analysis (PCA) separately (**Figure 5**). Root density and architectural traits (i.e., root length density, RLD; root biomass, RB; root surface area, RSA; root volume, RV; root tip number, RT; root fork number, RF; root crossing number, RC; and root-shoot ratio, R/S) and root morphology (i.e., root mean diameter, RMD; specific root length, SRL). The first principal component (PC1) extracted from this analysis was defined as the Root Morphology Index (RMI), which serves as a comprehensive quantitative metric to characterize root growth and development status under different tillage practices (**Figure 5A**). Similarly, for soil moisture conditions, principal component analysis was applied to soil moisture monitoring data collected from the seedling belt and straw mulch belt under different tillage practices across three key growth stages under different tillage practices. The principal component (PC1) derived from this analysis was defined as the Soil Moisture Content Index (SMCI), which comprehensively reflects the overall dynamic changes of soil moisture throughout the maize growing season (**Figure 5B**).

**Figure 6:**
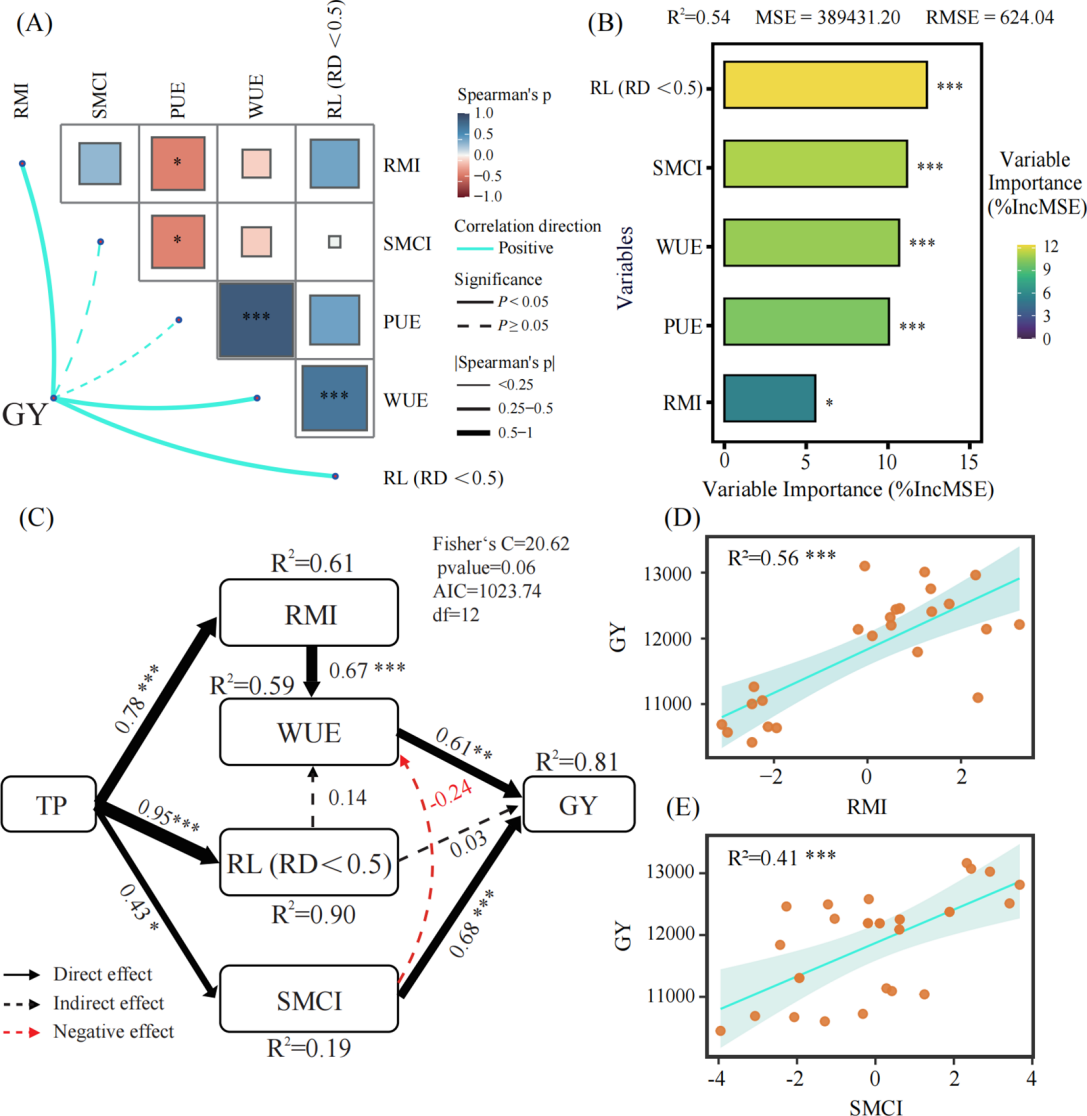
Multivariate analysis of maize grain yield and various indices. **(A)** Spearman’s correlation test results for the measured traits. **(B)** Variable importance from random forest analysis. **(C)** Structural equation model (SEM) showing the direct and indirect effects of tillage practices (TP) on grain yield (GY). In panel (C), black solid arrows indicate direct effects; black dashed arrows indicate indirect effects; red dashed arrows indicate negative effects. **(D)** Linear regression of grain yield (GY) on root morphology index (RMI). **(E)** Linear regression of grain yield (GY) on soil moisture content index (SMCI). Abbreviations in the figure: TP, tillage practices; RMI, root morphology index; WUE, water use efficiency; RL (RD < 0.5), root length (root diameter, RD < 0.5 mm); SMCI, soil moisture content index; GY, grain yield. *p < 0.05; **p < 0.01; ***p < 0.001; ns, no significant difference.

To identify the key driving factors influencing maize grain yield under different tillage practices, we employed Spearman’s test and random forest analysis for quantitative characterization (**Figures 6A, B**). Results of the Spearman’s test (**Figure 6A**) indicated that grain yield (GY) was significantly positively correlated with RL (RD < 0.5 mm), WUE, and RMI. Random forest (RF) analysis further quantified the relative importance of the aforementioned factors to grain yield (**Figure 6B**). Results indicated that RL (RD < 0.5 mm), SMCI, WUE, PUE, and RMI (*p* <0.001 or *p* <0.05) were the core variables for predicting grain yield, ranking among the top in terms of relative importance.

Based on the results of the above two analyses, we ultimately chose four core driving factors—RL (RD < 0.5 mm), RMI, SMCI, and WUE—and incorporated them into a structural equation model (SEM) to quantitatively dissect their direct and indirect influence pathways on GY. The structural equation model results clearly revealed two key regulatory pathways governing GY (**Figure 6C**): Pathway 1 (Soil Moisture-Dominated Pathway): tillage practices (TP) directly exerted a significant impact on grain yield (GY) by regulating the soil moisture content index (SMCI); Pathway 2 (Root Pathway): TP shaped the Root Morphology Index (RMI), thereby improving WUE and ultimately positively regulating GY. Additionally, linear regression analysis further verified that GY exhibited a significant positive correlation with both RMI (**Figure 6D**) and SMCI (**Figure 6E**), providing direct empirical support for the regulatory pathways revealed by the SEM.

## 4 Discussion

During the two experimental years (2023–2024), maize growth and yield exhibited clear responses to tillage practices under rain-fed conditions in the black soil region of Northeast China. Although interannual climatic variability influenced overall productivity, conservation tillage practices (no-tillage and modified strip-tillage) consistently enhanced crop growth performance compared with conventional tillage, improved ear traits, and increased grain yield. These improvements were closely associated with greater soil moisture content and soil water storage throughout the growing season, particularly in the 0–40 cm soil layer. Among the conservation tillage treatments, modified strip-tillage generally showed greater advantages in reducing growing-season water consumption and improving water use efficiency, indicating that localized soil disturbance combined with straw mulching can better coordinate soil water conservation with crop growth under rain-fed conditions.

### 4.1 Effects of tillage practices on yield traits

Dry matter serves as the fundamental material basis for crop growth and yield formation; therefore, enhancing the total dry matter accumulation represents a core agronomic strategy to improve grain yield potential (Jurgens et al., 1978). In the present study, grain yield was significantly higher under no-tillage and modified strip-tillage than under conventional tillage. This can presumably be attributed to the improvements in soil water retention capacity and fertility induced by conservation tillage (Li et al., 2024), which in turn promote leaf area expansion, mitigate leaf senescence (Cheng et al., 2025), and extend the effective photosynthetic duration — conditions that are directly favorable for biomass accumulation and subsequent grain yield formation. In addition, conservation tillage practices (no-tillage and modified strip-tillage) resulted in significantly higher kernel number per ear (attributed to increased ear length and kernel number per row) and 100-kernel weight compared with conventional tillage. Our findings align with those reported by (Wang H. et al., 2022), who demonstrated that conservation tillage combined with straw mulching effectively improves soil moisture status and extends the grain-filling period, thereby facilitating the translocation of photosynthetic assimilates to grains and ultimately increasing the yield components.

### 4.2 Effects of tillage practices on root traits – root density and architectural traits

As the core interface between crops and the soil environment, root traits determine the acquisition efficiency of soil water and nutrients (Han et al., 2017, 2022). In the present study, root development of maize was substantially improved under conservation tillage of no-tillage and modified strip-tillage, compared with conventional tillage, indicating reduced soil disturbance and straw mulching promote the development of more complex and highly branched root systems. Conventional tillage results in soil conditions characterized by low pore connectivity and limited displacement space for soil particles (Suzuki et al., 2013), which inhibits root growth. In contrast, conservation tillage reduces soil bulk density, increases soil porosity, and particularly enhances the proportion of microporosity (0.5–1.0 mm) in the 10–20 cm soil layer (Hernandez-Ramirez et al., 2014), thereby creating a low-resistance farmland environment conducive to root growth. These findings are consistent with the previous study (Gao et al., 2023), as well as the latest research of Han et al. (2025).

Root length density and root biomass are good density-based indicators to determine the spatial topological structure of plants’ spatial occupancy in soil (Cabal et al., 2021). In this study, both root length density and root biomass were found significantly higher under no-tillage and modified strip-tillage than those under conventional tillage (*p* < 0.05), which is likely attributed to the multi-year conservation tillage of no-tillage and modified strip-tillage improving soil porosity, aeration, and organic matter content. This creates a looser soil physical environment for root growth under rainfed conditions, which favors greater biomass allocation to roots and thereby enhances root density per unit soil volume (i.e., root length density and root biomass) (Wang et al., 2015). Additionally, root surface area and root volume in modified strip-tillage treatment were 48.89%–50.18% and 58.76%–65.74% higher than that in the conventional tillage treatment. This phenomenon may be attributed to the fact that conservation tillage improves soil structure, which in turn promotes root hair development, epidermal cell proliferation, and other physiological processes (Wan et al., 2023). These changes enhance the development of root absorptive structures, enabling roots to absorb water from deep soil layers and translocate it to the aboveground organs (Fu et al., 2025).

### 4.3 Effects of tillage practices on root morphology and root diameter classes

Differences in root mean diameter are directly associated with the lignification degree and resource allocation strategy of maize roots. Previous studies have demonstrated that the respiratory consumption per unit root length of thicker roots is 18%–25% higher than that of thinner roots (Suslov et al., 2024). Under water stress conditions, crops exhibit an adaptive regulatory strategy characterized by “thickening of surface roots and thinning of deep roots”—surface roots reduce water loss through lignification (Protto et al., 2024), while deep roots minimize metabolic costs for water acquisition by reducing their diameter. However, in the present study, in the root mean diameter of maize under no-tillage and modified strip-tillage treatments was significantly lower than that under conventional tillage by 12.57%–19.38% and 13.77%–26.25%, respectively. This result is attributed to the fact that no-tillage and modified strip-tillage treatments under rain-fed conditions ensure adequate water availability for maize growth, satisfying the water requirements of the root system and thereby reducing root mean diameter. Specific root length reflects the elongation capacity and resource allocation strategy of roots per unit biomass (Ostonen et al., 2007). According to the same study, drought treatment resulted in a decreasing trend in specific root length (i.e., by 11%), while irrigation treatment led to an increasing trend in specific root length (i.e., by 8%), although neither treatment reached statistical significance (Ostonen et al., 2007). Roots with higher specific root length are usually fine roots with a diameter less than 0.5 mm, which are highly beneficial for improving water and nutrient uptake. In the present study, the value of specific root length under three tillage treatments followed the order of Modified strip-tillage > No-Tillage > Conventional Tillage, although the differences among treatments were not significant (*p* > 0.05). These findings are consistent with those reported by Dong et al. (2024).

Roots of different diameter perform distinct functional roles in water and nutrient acquisition — which can alter based on the soil structure-altering management (Han et al., 2016). In general, fine roots with diameters of < 0.5 mm are characterized by high sensitivity to soil moisture and exhibit strong absorptive capacity (Leuschner et al., 2001). Roots measuring 0.5–2.0 mm serve as the primary region for water and nutrient absorption, the absorption efficiency gradually decreases as diameter increases (Dorlodot et al., 2007); roots in the 2.0–5.0 mm range function as a transitional zone, primarily undertaking mechanical support and nutrient translocation while also fulfilling partial absorption functions (Li et al., 2024). In the present study, random forest analysis (**Figure 6B**) indicates that root length of fine roots (root diameter, RD < 0.5 mm) is critical for yield enhancement. Consistent with this finding, compared with the conventional tillage treatment, the no-tillage and modified strip-tillage treatments increased root length (RD < 0.5 mm) by 16.30%–39.54% and 93.23%–95.22%, respectively. These increases in fine root length are associated with soil penetration resistance; such alterations in root traits can be attributed to conservation tillage mitigating soil compaction effects, thereby modifying the proportion of root length across different diameter classes (Yin et al., 2024). Structural equation modeling (SEM) analysis (**Figure 6C**) further confirmed that tillage practices (TP) exerted an extremely significant positive effect on RL (RD < 0.5 mm) (*p* < 0.001), with a path coefficient of 0.95 and an R² of 0.90—indicating strong explanatory power for the variation in fine root length.

### 4.4 Effects of tillage practices on soil moisture and water use efficiency

In rain-fed agricultural areas, water is the key limiting factor for crop growth (Zhang et al., 2015). Therefore, improving farmland water use efficiency is of great practical significance for ensuring crop productivity (Li et al., 2025). In contrast, conventional tillage in Northeast China severely disturbs the soil, disrupting the native farmland soil structure. Although conventional tillage loosens the surface soil, raises soil temperature in alpine regions, and promotes early-stage maize growth, it simultaneously expands the soil-air contact area—thus accelerating soil water loss via evaporation, an effect particularly pronounced in the topsoil (Colombi et al., 2015; Humberto and Ruis, 2018). The conventional tillage system in Northeast China involves a series of operational processes, including straw removal, rotary tillage for land preparation, ridge formation, and fertilization. These operations significantly increase the contact area between soil and air, accelerating soil water evaporation rate and thereby reducing soil water storage capacity and water use efficiency (Huo et al., 2024; Zhang et al., 2024). In contrast, the no-tillage and modified strip-tillage treatments in this study exhibit dual water-preserving effects via straw mulching and no-tillage/reduced tillage practices (Zhang et al., 2024). On the one hand, no-tillage and modified strip-tillage promote the formation of soil microporosity, enhance soil water infiltration, and ultimately improve soil water-holding capacity as well as water availability throughout the entire maize growth period. This constitutes the core mechanism underlying the increased soil water content and enhanced soil water storage capacity observed in the no-tillage and modified strip-tillage treatments in the present study. The finding is also consistent with previous research (Dou et al., 2024; Sun et al., 2024).

The present study further found that compared with conventional tillage, soil water storage under no-tillage and modified strip-tillage treatments increased by 5.91% and 6.86% before sowing, and by 6.37% and 7.70% after harvest, respectively. This further confirms that conservation tillage exhibits advantages in improving soil water storage capacity (Zhang et al., 2022). Generally, farmland soil water consumption comprises inter-row soil evaporation and crop transpiration, with inter-row soil evaporation being an ineffective soil water loss (Du et al., 2022). Reducing such ineffective loss (e.g., inter-row soil evaporation) is an effective approach to improve crop water use efficiency. In the present study, the modified strip-tillage treatment reduced total water consumption by 1.95%–2.41% relative to conventional tillage, while water use efficiency increased by 14.34%–18.48% under no-tillage and 16.33%–18.27% under modified strip-tillage, respectively. This finding is consistent with previous studies demonstrating that straw mulching (a key component of conservation tillage) can effectively preserve soil moisture and reduce inter-row soil evaporation in arid regions (Zhang et al., 2022), thereby reducing ineffective farmland soil water loss and total water consumption during the crop growth period (Hussain et al., 2021). These results indicate that adopting conservation tillage practices in the black soil region of Northeast China positively enhances maize water use efficiency.

### 4.5 Synthesis: relationships between root characteristics and soil water dynamics

The combined results of principal component analysis (PCA), random forest, and structural equation modeling (SEM) indicate that conservation tillage regulates maize yield through coordinated effects on soil water availability and root system traits. Straw mulching and reduced soil disturbance enhance soil moisture storage and reduce ineffective evaporation, while improved soil physical conditions promote favorable root morphological and root density traits (Du et al., 2022; Zhang et al., 2022; Zhang et al., 2024). Although modified strip-tillage and no-tillage differed significantly in root characteristics, grain yield and water use efficiency were similar, with modified strip-tillage performing slightly better overall. Since this experiment was conducted in the high-latitude region of Northeast China, no-tillage with straw mulch may result in relatively low soil temperature and poor seedling emergence in spring (**Supplementary Figure**). In contrast, modified strip-tillage concentrates straw in inter-row fallow zones, which significantly increased seedbed temperature and seedling establishment, thereby exhibiting stronger production adaptability to local production conditions (Chen et al., 2021; Zhang et al., 2024; Liu et al., 2025). The underlying mechanism for the divergent root traits between the two tillage practices lies in their substantial differences in land preparation and straw distribution, despite both being straw mulch-based conservation tillage methods. No-tillage involves no pre-sowing tillage and full surface straw mulch, which benefits water retention but increases seedbed compaction, thereby restricting early root growth (Humberto and Ruis, 2018). In contrast, modified strip-tillage, applies 5–7 cm shallow rotary tillage in the seedbed zone while retaining straw between rows. This practice reduces compaction, optimizes soil temperature and moisture conditions, creates a more favorable environment for root growth, and promotes fine-root development and root architectural optimization (Zhang et al., 2015; Zhang et al., 2024). Consequently, the two tillage practices differ significantly in seedbed soil temperature, which is positively correlated with early maize root growth (Zhang et al., 2024). Such differences in soil physical environment and tillage practice drive the divergence in root trait and explain why yield remains similar despite distinct root development patterns (Dou et al., 2024; Yin et al., 2024).

We speculate that modified strip-tillage improves water use efficiency and yield primarily by optimizing root morphology to enhance water uptake, whereas no-tillage relies more on straw mulching for soil water conservation. These differing mechanisms for improving yield and WUE help explain the significant differences in root traits but non-significant yield differences between the two tillage systems. Modified strip-tillage integrates strong soil water conservation with enhanced fine-root growth in a favorable seedbed environment. This dual regulation of soil water status and root architecture supports its superior performance in improving maize WUE and grain yield under rain-fed conditions in the black soil region of Northeast China (Chen et al., 2021; Sha et al., 2024; Yin et al., 2024). Therefore, the linkages among soil temperature, soil moisture, and root growth deserve further investigation in future research. In addition, since roots were only sampled at the flowering stage in the present study, the dynamics of root growth across other key growth stages remain unclear and should be systematically examined to reveal temporal variation patterns.

### 4.6 Agronomic and scientific implications

From an agronomic perspective, the results demonstrate that modified strip-tillage is a regionally appropriate conservation tillage strategy for maize production in the cold, rain-fed black soil region of Northeast China. By combining localized soil disturbance in the seedbed with inter-row straw mulching, modified strip-tillage enhances soil moisture storage, reduces ineffective soil evaporation, and improves water use efficiency and grain yield, while avoiding the delayed emergence and early-season stress often associated with full no-tillage systems (Chen et al., 2021; Zhang et al., 2022; Wang et al., 2024; Zhang et al., 2024). These advantages suggest that modified strip-tillage can help stabilize maize production under interannual rainfall variability and support sustainable water and soil management in this region (Wang Z. et al., 2022; Rui et al., 2025).

From a scientific perspective, this study advances understanding of how conservation tillage regulates crop performance by explicitly linking soil water dynamics with root system traits. The findings show that improvements in yield and water use efficiency under conservation tillage are driven not only by enhanced soil water availability, but also by qualitative adjustments in root morphology and architecture—particularly fine-root development and root branching—rather than by increased root biomass alone (Zhang et al., 2022; Yin et al., 2024). This root-centered mechanism complements existing soil-focused studies (Dou et al., 2024; Sun et al., 2024) and highlights the importance of integrating root traits into future evaluations of conservation tillage systems and soil–crop–water interactions.

## 5 Conclusion

In the rain-fed black soil region of Northeast China, conservation tillage significantly improved maize yield and water use efficiency compared with conventional tillage. Both no-tillage and modified strip-tillage increased soil moisture storage and reduced ineffective water loss, resulting in yield increases of 11.89%–17.15% and substantial improvements in water use efficiency. Conservation tillage also markedly altered maize root system traits, enhancing root length, branching, and fine-root development while reducing mean root diameter. Among the tested practices, modified strip-tillage showed greater advantages in promoting fine-root proliferation and improving water use efficiency. Overall, modified strip-tillage represents an effective conservation tillage strategy for improving maize productivity and water use efficiency in cold, rain-fed black soil regions.

## Data Availability

The raw data supporting the conclusions of this article will be made available by the authors, without undue reservation.
