# Supplementary material for: Modified strip-tillage boosts maize grain yield and water use efficiency by enhancing fine root length in Northeast China’s black soil
Source: Front Plant Sci. 2026 Apr 23;17:1809158. doi: 10.3389/fpls.2026.1809158 (PMC13149157; doi:10.3389/fpls.2026.1809158)
Supplement: Supplementary file 1 [file DataSheet1.pdf]

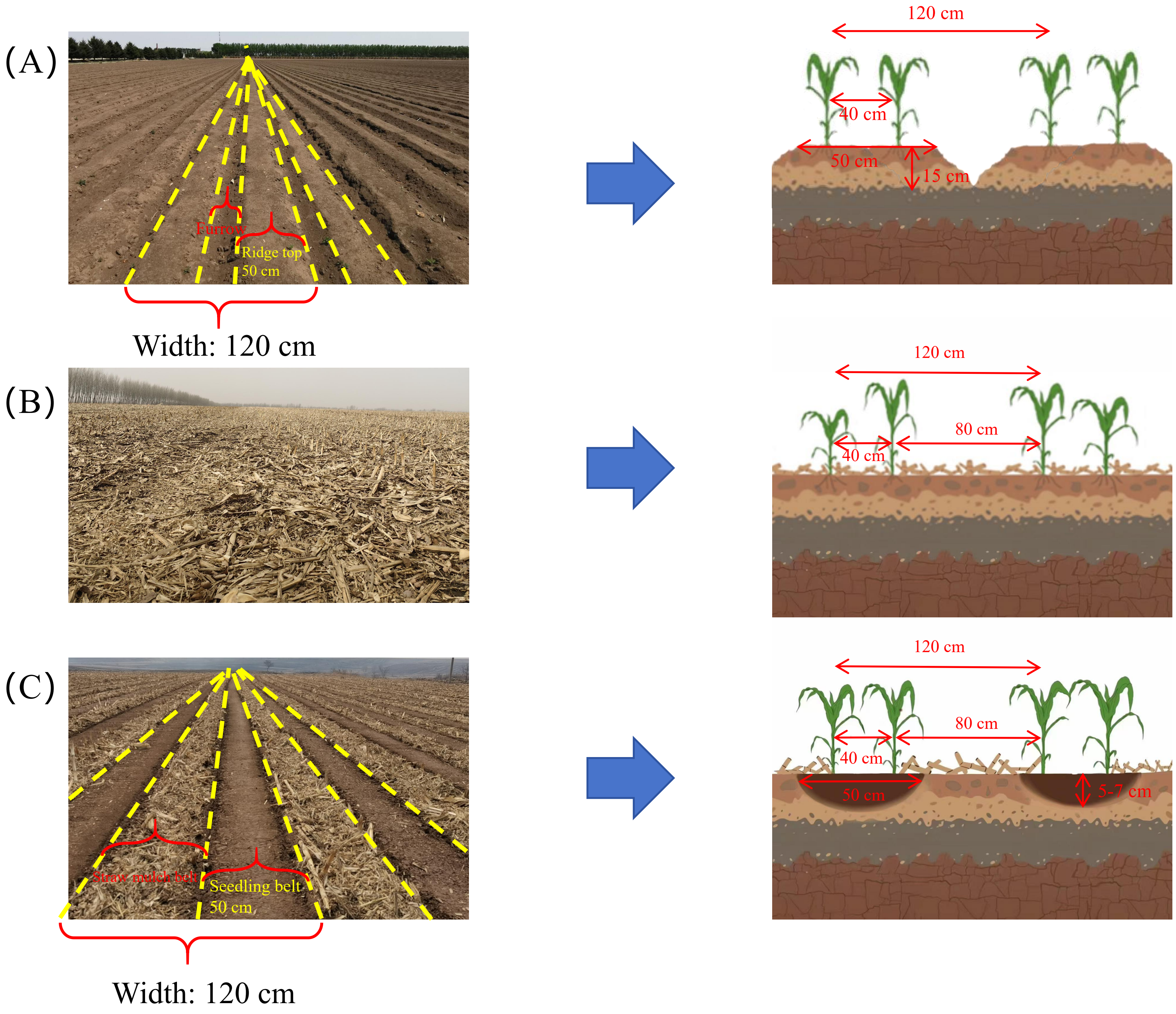

Figure 1 Schematic siagram of different tillage practices.
(A) Conventional tillage;
(B) No-tillage;
(C) Modified strip-tillage.
